# Pain after upper limb surgery under peripheral nerve block is associated with gut microbiome composition and diversity

**DOI:** 10.1016/j.ynpai.2021.100072

**Published:** 2021-08-18

**Authors:** David Brenner, Paul Cherry, Tim Switzer, Ihsan Butt, Catherine Stanton, Kiera Murphy, Brian McNamara, Gabriella Iohom, Siobhain M. O'Mahony, George Shorten

**Affiliations:** a Department of Anesthesia and Intensive Care Medicine, Cork University Hospital and University College Cork, Ireland; b Teagasc Food Research Centre, Moorepark, Fermoy, County Cork, Ireland; c APC Microbiome Ireland, University College Cork, Ireland; d Department of Clinical Neurophysiology Cork University Hospital, Ireland; e Department of Anatomy and Neuroscience University College Cork, Ireland

**Keywords:** Gut microbiota, Nerve block, Rebound pain, Regional anaesthesia

## Abstract

Gut microbiota play a role in certain pain states. Hence, these microbiota also influence somatic pain. We aimed to determine if there was an association between gut microbiota (composition and diversity) and postoperative pain. Patients (n = 20) undergoing surgical fixation of distal radius fracture under axillary brachial plexus block were studied. Gut microbiota diversity and abundance were analysed for association with: (i) a verbal pain rating scale of < 4/10 throughout the first 24 h after surgery (ii) a level of pain deemed “acceptable” by the patient during the first 24 h following surgery (iii) a maximum self-reported pain score during the first 24 h postoperatively and (iv) analgesic consumption during the first postoperative week. Analgesic consumption was inversely correlated with the Shannon index of alpha diversity. There were also significant differences, at the genus level (including *Lachnospira)*, with respect to pain being “not acceptable” at 24 h postoperatively. *Porphyromonas* was more abundant in the group reporting an acceptable pain level at 24 h. An inverse correlation was noted between abundance of *Collinsella* and maximum self-reported pain score with movement. We have demonstrated for the first time that postoperative pain is associated with gut microbiota composition and diversity. Further work on the relationship between the gut microbiome and somatic pain may offer new therapeutic targets.

## Highlights

• Postoperative pain is associated with gut microbiota composition and diversity.
• Analgesic consumption is inversely correlated with alpha diversity.
• Further work on the relationship between the gut microbiome and somatic pain may offer new therapeutic targets.

## 1 Introduction

There is growing appreciation for the importance of the gut microbiota in health and disease. Gut microbiota can influence the bidirectional signalling pathways between the central nervous system (CNS) and gastrointestinal (GI) tract, termed the microbiota-gut-brain axis (Butler et al., 2019, Felice and O’Mahony, 2017, Guo et al., 2019). Disturbances in this axis have been associated with several disease states (Guo et al., 2019) including stress-related disorders such as anxiety and depression (Kelly et al., 2016), fibromyalgia (Malatji et al., 2019), migraine (Tang et al., 2019), as well as GI disorders such as irritable bowel syndrome (IBS) (O’ Mahony et al., 2017O’ Mahony et al., 2017, Pittayanon et al., 2019). IBS patient cohorts demonstrate distinct gut microbial taxa compared to healthy controls (Pittayanon et al., 2019). Furthermore, differences in diversity and specific bacterial species are associated with symptom severity in chronic pelvic pain syndrome (Shoskes et al., 2016). Microbial manipulation such as prebiotic and probiotic administration, as well as faecal microbiota transplantation (FMT), have decreased visceral hypersensitivity in pre-clinical models (Bai et al., 2018, Luczynski et al., 2017, Verdú et al., 2006). Moreover, the strain *Bifidobacterium breve* NCIMB 702,258 is reported to increase endocannabinoid (EC) levels in the liver and epididymal adipose tissue of mice (Patterson et al., 2017). These findings indicate that specific manipulation of the gut microbiota may elicit an analgesic effect (Guo et al., 2019).

Although it has not been thoroughly investigated in humans, there is preclinical data available to support the relationship between gut microbiota and somatic pain (Amaral et al., 2008). Furthermore, some of the pathways and regulators of visceral pain and hypersensitivity are also critical in somatic pain handling (Jänig, 2014, Lovick, 2016, Salaga et al., 2016, Sharkey and Wiley, 2016). These include peripheral and central sensitization, and alteration of descending inhibitory pathways. These neuroplastic changes have been well documented in the examination of persistent post-surgical pain (Gerbershagen, 2013). Moreover, somatic pain is influenced by changes in immune and stress responses (Chapman et al., 2008), both of which are influenced by the gut microbiota (Codagnone et al., 2019).

Rebound pain (RP) is a quantifiable difference in pain scores between that elicited when a nerve blockade is effective, and that elicited after the blockade has resolved (Williams et al., 2007). RP may represent a manifestation of neural hypersensitivity, and offer an accessible clinical model suitable for examining the association between the gut microbiota and perioperative neuroplastic changes.

The primary objective of this study was to determine the association (if any) between microbiota diversity (Clarke et al., 2014, Lapthorne et al., 2013) and the magnitude and characteristics of pain after offset of peripheral nerve block (PNB) in patients who have undergone upper limb surgery.

Secondary objectives were:

To determine associations (if any) between relative abundance of microbial taxa and other characteristics of postoperative and rebound pain, and postoperative analgesic consumption.
To describe (post PNB) rebound pain by quantifying its clinical, psychological and neurophysiological characteristics in this patient cohort.

## 2 Methods

With Institutional Ethical approval (Clinical Research Ethics Committee of Cork Teaching Hospitals, Cork, Ireland - ECM4(w)11/10/16; 15 November 2016, Chairperson Prof M.G. Molloy] and having obtained written informed consent from each, 20 ASA I-II patients scheduled to undergo upper limb surgery under axillary brachial plexus block (ABPB) were recruited. The study was conducted at the Department of Anaesthesia and Intensive Care Medicine, Cork University Hospital, Ireland. The trial was registered at ClinicalTrials.gov (NCT02998177; 15 December 2016).

### 2.1 Inclusion criteria

Age 18–80 years; patients undergoing fixation of distal radius fracture (ORIF or K-wiring) under ABPB.

### 2.2 Exclusion criteria

Contraindication to regional anaesthesia, uncontrolled pain (Verbal Rating Score (VRS; 0–10) ≥ 5 at rest despite adequate analgesic measures); chronic pain syndrome; history of peripheral neuropathy; pre-existing nerve damage in the operative arm; axillary surgery in the past; cognitive impairment (MiniMental State Score < 24); language barrier; depression; diabetes; obesity (BMI > 35); antibiotic therapy in the preceding 30 days; recent (<1 year) administration of probiotics.

### 2.3 Perioperative management

Preoperative pain levels and total analgesic consumption were recorded in a pain diary. All patients underwent ultrasound guided ABPB. Local anaesthetic mixture containing lidocaine 2% + adrenaline 1:200.000 and bupivacaine 0.5%, 10 ml each, was applied to each of the four nerves. Cefuroxime 1.5 G i.v. was administered immediately preoperatively according to hospital guidelines. Patients received i.v. diclofenac 75 mg in the operating theatre; opioids and dexamethasone were not administered.

### 2.4 Postoperative assessment

*Block assessment:* sensory and motor function for each of the ulnar, median, radial, musculocutaneous nerves were assessed and recorded immediately after arrival to the recovery room.

*Self-report of pain (VRS, 0*–*10)* was recorded postoperatively commencing on the patient’s arrival to the recovery room and during the first postoperative week. Rebound Pain Score (RPS) was defined as the difference between maximum and minimum VRS (only if the block was successful). Rebound pain was present by definition if RPS > 3. Patient’s self-reported acceptable pain level (VRS, 0–10) was recorded.

A *short form McGill Pain Questionnaire* was completed by the patient from immediately after arrival to the recovery room and thereafter during the first 24hrs postoperatively if the patient detected a significant change in pain intensity and/or quality.

Each patient’s analgesic consumption was recorded in a *pain diary* during the first postoperative week.

### 2.5 Postoperative analgesia

Paracetamol 1 g (QDS), diclofenac 75 mg (BD) and oxycodone 10 mg (modified release, BD) were administered regularly to all patients. Oxycodone 5–10 mg (fast release) four hourly was administered for breakthrough pain, at the patient’s request.

### 2.6 Neurophysiological assessments

Bilateral electronic quantitative sensory testing (QST) was performed pre- and postoperatively (after complete offset of the block, within the first 24 h after block placement) by a trained investigator. Sensory threshold (ST), pain perception threshold (PPT) and pain tolerance threshold (PTT) were assessed in each patient using a Nihon Kohden Neuropack S1 EMG/EP stimulator. ST, PPT and PTT was recorded using the staircase method (1 mA ramping), and a standardized technique in the forearm/hand (C5-T1 dermatomes) of the affected and contralateral upper limbs.

### 2.7 Faecal sampling

Participants were given collection packs and detailed instructions on how to collect their preoperative faecal samples (on the day of /or day before surgery; where feasible) and the first sample after surgery. The samples were refrigerated before transport and then maintained at -80°C until analysis.

### 2.8 DNA extraction

Microbial DNA was extracted from 0.1 g stool samples using the FastDNA® Spin Kit (MP Biomedicals, Santa Ana, CA, USA).

### 2.9 16S rRNA amplicon sequencing

16S rRNA amplicon sequencing was conducted using the Illumina MiSeq platform. 16S rRNA sequencing library preparation was completed following the 16S metagenomic sequencing library protocol (Illumina). Genomic DNA was amplified using primers specific to the V3-V4 hypervariable region of the 16S ribosomal RNA gene (Forward primer 5′ TCGTCGGCAGCGTCAGATGTGTATAAGAGACAGCCTACGGGNGGCWGCAG; Reverse primer 5′ GTCTCGTGGGCTCGGAGATGTGTATAAGAGACAGGACTACHVGGGTATCTAATCC).. PCR products were visualised using gel electrophoresis (1X TAE buffer, 1.5% agarose, 100 V) and successful PCR products were cleaned using AMPure XP magnetic beads (Labplan, Dublin, Ireland). A second PCR reaction was completed on 5 µl of the purified DNA. Two indexing primers (Illumina Nextera XT indexing primers, Illumina, Sweden) were used per sample to provide a unique index and facilitate sample pooling for sequencing on a single flow cell and demultiplexing prior to analysis. Each PCR reaction contained 5 µl index 1 primer (N7xx), 5 µl index 2 primer (S5xx), 25 µl 2x Kapa HiFi Hot Start Ready mix, and 10 µl PCR grade water. PCRs conditions were as described above, with only 8 amplification cycles. PCR products were visualised and cleaned as described above. Samples were quantified using the Qubit 3 fluorometer (Bio-Sciences, Dublin, Ireland) and samples were pooled to an equimolar mix. The samples were sequenced using a 2 × 250 cycle kit, following standard Illumina sequencing protocols.

### 2.10 Bioinformatics and statistical analysis

Collected continuous data was examined for normality. Between group comparisons (e.g. presence of rebound pain) of quantitative data relating to patients (e.g. VRS, pain thresholds) were examined using unpaired Student-t tests with Bonferroni correction. Categorical data were examined using the Chi-squared test or Fisher’s Exact test as appropriate. Correlation between continuous variables was examined using Pearson’s correlation coefficient. P < 0.05 was considered significant.

All samples had > 52,000 reads. Data were analysed as per the following biological conditions (groupings): Group 1 (pain level acceptable to patient - first 24 h: Yes vs No) and Group 2 (VRS max < 4 - first 24 h: Yes vs No).

Paired-end reads were assembled using FLASH (Magoc and Salzberg, 2011). Further processing of paired-end reads including quality filtering based on a quality score of > 25 and removal of mismatched barcodes and sequences was completed using QIIME version 1.9.0. Denoising, chimera detection and clustering into OTU grouping were performed using USEARCH v746. OTUs were aligned using PyNAST and taxonomy was assigned using BLAST against the SILVA SSURef database release 123. Statistical analysis was performed using the Calypso online software (version 8.68). All samples had > 52,000 reads. Taxa present at < 0.01% were removed and up to 20,000 taxa are included in the analysis, unless otherwise stated. Cumulative-sum scaling was performed to account for the non-normal distribution of taxonomic count data. Alpha diversity was measured using Shannon diversity, evenness, Chao1, Simpsons Index and Observed species. Beta diversity was measured based on Principal coordinate analysis of Bray–Curtis distance matrices. A Permutational multivariate analysis of variance (PERMANOVA) was used to determine statistical differences of beta diversity. Differential abundance between biological conditions was determined using linear discriminant analysis (LDA) effect size (LEfSe) (Segata et al., 2011). Spearman correlation was conducted to examine potential associations between the environmental variables Maximum pain score with movement (first 24 h), and analgesic consumption (morphine equivalence, first week), with bacterial abundance (genus level taxonomy).

## 3 Results

We recruited twenty patients between February and May 2017 into this prospective, observational study. Patient characteristics are summarised in Supplementary Table 1. No patients were excluded, but data from two patients have not been included in the gut microbiota analysis due a breach of protocol in sample collection and storage. An insufficient number of faecal samples (5/20) were obtained preoperatively to justify a separate analysis of change in microbiome composition. The patient flowchart is illustrated in Supplementary Figure 1.

### 3.1 Clinical results

**Fig. 1:**
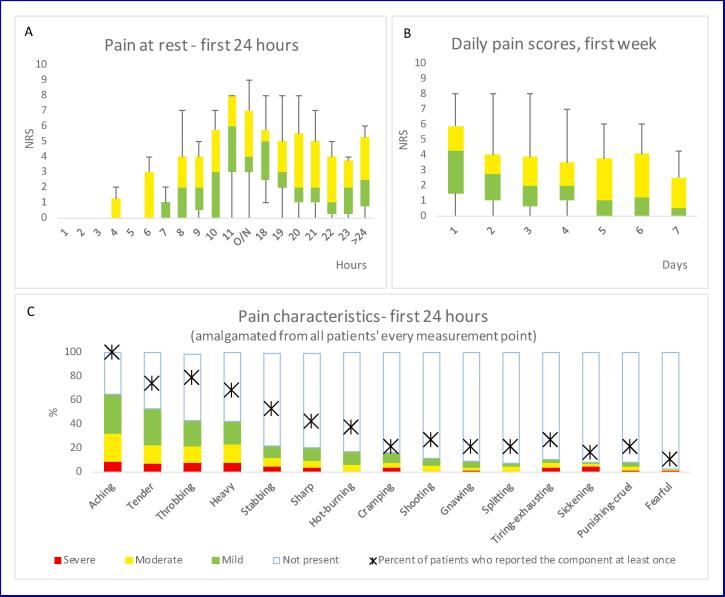
Summary of the postoperative pain experience. (A) Pain scores at rest during the first 24 h postoperatively. (B) Daily pain scores during the first postoperative week. (C) Summary of the Short Form McGill pain Questionnaire results: columns are showing the severity distribution of the certain pain component (amalgamated from every patients’ every measurement point); the percentage of patients who reported the certain component at least once during the first 24 h postoperatively is marked by asterisks. Data are expressed in median and interquartile range or percentage, as appropriate. NRS, Numerical Rating Scale; O/N, overnight.

On the day before surgery, the patients’ VRS for pain at rest was 4.5 (2.59) [mean (SD)]. Every block was successful for the purposes of surgical anaesthesia; no block-related adverse events occurred. Block regression started and completed in 6.68 (2.88) and 12.21 (3.7) hours, respectively (mean; SD). Seventeen of the 20 patients experienced RP after resolution of ABPB. The mean (SD) RPS reported was 5.4 (3.11). No significant correlation was identified between preoperative VRS and RPS (CC = -0.27), and magnitude of RP and age (CC = 0.15). Patient self-report of pain and analgesic consumption was characterised by great variation and neither demonstrated significant association with the type of the surgery (ORIF vs K-wiring). For 8/20 patients the pain scores (VRS 0–10) over the first 24 h postoperatively were all less than their acceptable pain threshold. The pain characteristics for the first 24 h postoperatively are summarised in Fig. 1. The most common pain qualities reported were aching, throbbing, tender, heavy and stabbing. Twelve of nineteen patients reported paraesthesia (“pins and needles”) during block regression.

There was no significant correlation between cumulative analgesic consumption at 24 hrs postoperatively and magnitude of RPS (CC = 0.02). The adherence to analgesia protocol was similar amongst patients with or without RP.

Patients’ median daily pain scores for the first postoperative week are summarised in Fig. 1. The mean (SD) cumulative analgesic consumption (opioid equivalent) for the first postoperative week was 253.69 mg (107.04).

One month after surgery, three of the 20 patients were taking painkillers regularly for symptoms related to their surgery and only one was restricted in everyday activities as a result of his pain.

The mean (SD) time between block placement and postoperative QST measurements were 21.16 (3.82) hours. When comparing pre- and postsurgical QST results, surgical side PPT and PTT was greater postoperatively then preoperatively (p 0.023 and 0.025, respectively). No differences were observed between (i.) surgical vs control side parameters; (ii.) control side pre- vs postoperative parameters; (iii.) pre- vs postoperative somatosensory thresholds. Pre- and postoperative PPT and PTT were not different amongst patients with or without RP (Supplementary Table 2**).**

### 3.2 Gut microbiota

#### 3.2.1 Alpha diversity

There were no significant differences in Shannon index (overall taxa diversity), evenness, richness, Simpson’s diversity, and Chao1 within group 1-pain level acceptable within 24 h **(**Fig. 2a**).** However, evenness (abundance of taxa) was significantly less in patients who reported VRS < 4 during the first 24 h postoperatively **(**Fig. 2b**).**

**Fig. 2a:**
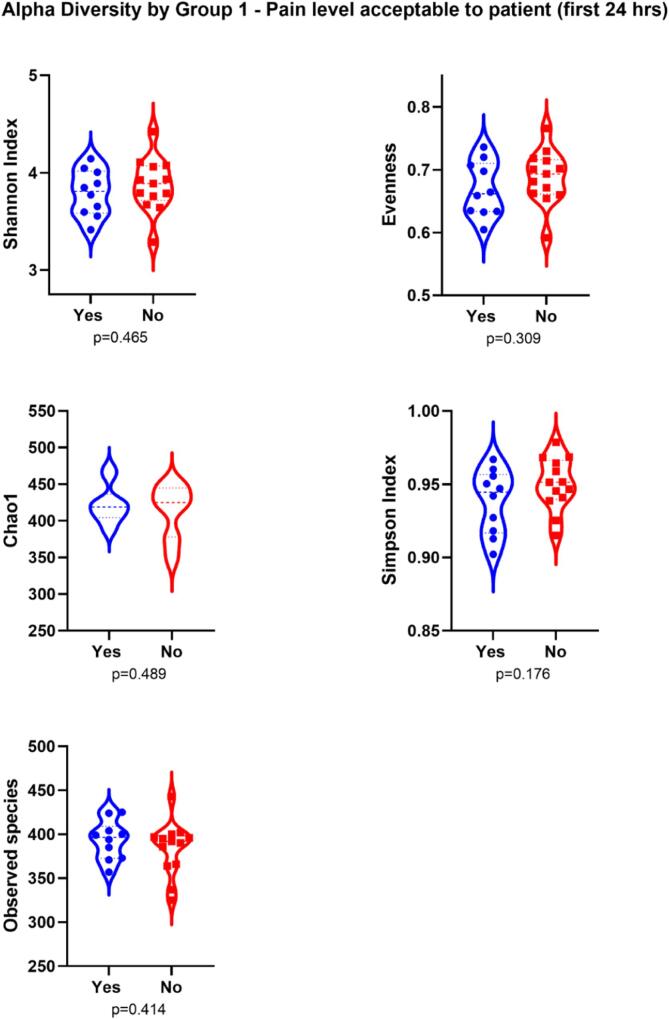
Violin plot showing Shannon Index, Evenness, Chao 1, Simpson’s diversity and richness metrics of alpha diversity, based on study groupings. Group 1 - Pain level acceptable to patient (first 24 hrs).

**Fig. 2b:**
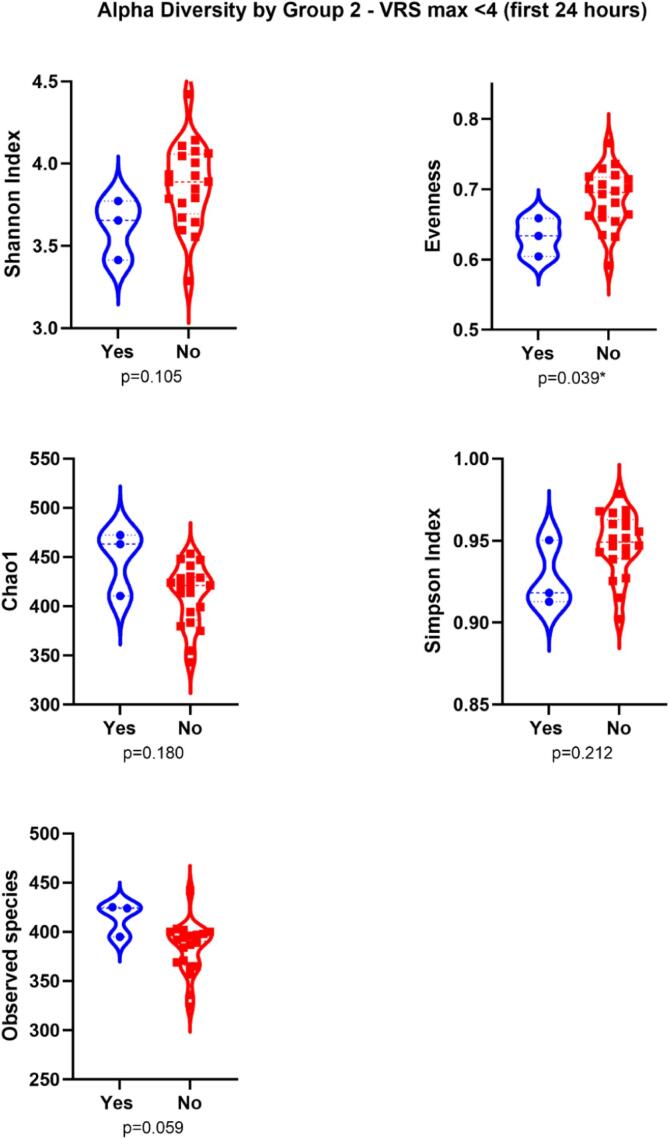
Violin plot showing Shannon Index, Evenness, Chao 1, Simpson’s diversity and richness metrics of alpha diversity, based on study groupings. Group 2 – Maximum verbal rating scale score (VRS max) < 4 (first 24 h). * *p* < 0.05 (independent T-test).

#### 3.2.2 Beta diversity

**Fig. 3:**
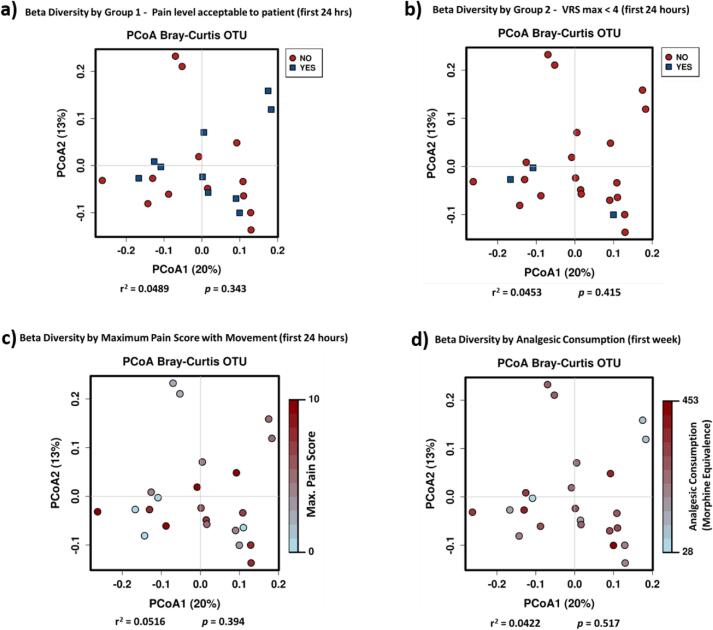
Principal coordinate analysis (PCoA) plots of Bray–Curtis dissimilarity distances (of operational taxonomic units), as a metric of Beta diversity. Statistical significance was determined using the Adonis analysis of variance function (Permutational Multivariate Analysis Of Variance Using Distance Matrices). **a.** Group 1 - Pain level acceptable to patient (first 24 hrs). **b.** Group 2 – Maximum verbal rating scale score (VRS max) < 4 (first 24 h). **c.** Maximum pain score with movement (first 24 h). **d.** Analgesic consumption (morphine equivalence, first week).

Beta diversity was not statistically significantly different between groups for maximum pain score with movement (first 24 h), or analgesic consumption **(**Fig. 3**).**

#### 3.2.3 Abundance

Abundance by phylum, family and genus from the stool samples analysed are summarised in Supplementary Figures 2 and 3.

#### 3.2.4 LEfSe analysis

**Fig. 4:**
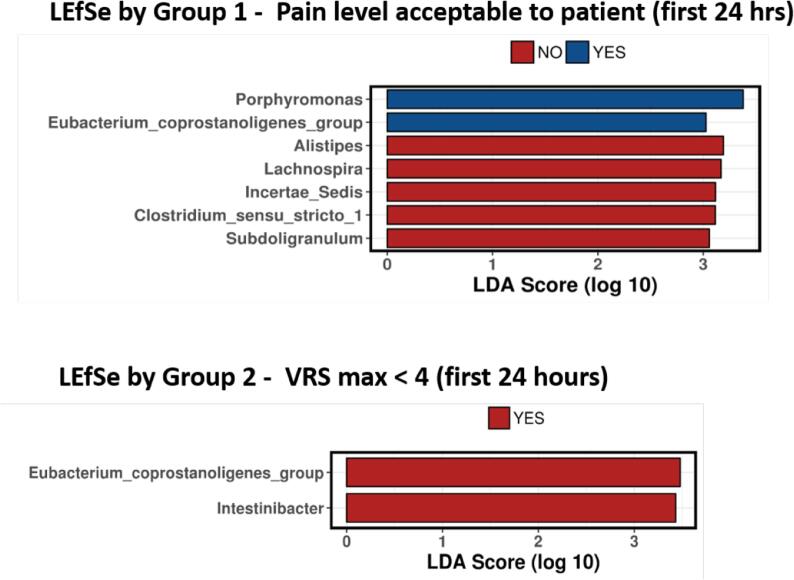
Histograms displaying LDA score following linear discriminant analysis effect size (LEfSe) analysis to show bacterial genera which are differentially abundant within groupings. Group 1 - Pain level acceptable to patient (first 24 hrs); Group 2 – Maximum verbal rating scale score (VRS max) < 4 (first 24 h).

LEfSe analysis was used to determine the discriminative bacteria (more abundant) most likely to explain difference between the groups. For Group 1, *Porphyromonas* and the *Eubacterium coprostanoligenes* group were discriminative bacterial genera in those who answered “YES”, whereas the genera *Alistipes*, *Lachnospira*, *Incertae Sedis*, *Clostridium sensu stricto 1*, and *Subdogranulum* were discriminative genera of those who answered “NO”. For Group 2, *Intestinibacter* and the *Eubacterium coprostanoligenes* group were discriminative bacterial genera of those who gave a VRS < 4. There were no differentially abundant bacterial genera for those who gave a VRS > 4 **(**Fig. 4**)**.

#### 3.2.5 Correlations with relative abundance of bacteria at genera level

**Fig. 5:**
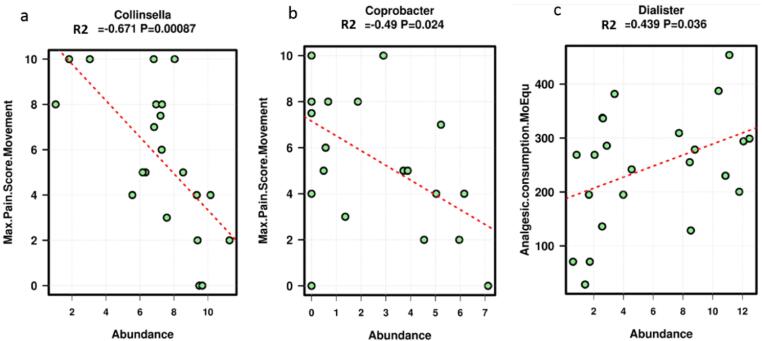
Statistically significant Spearman rank-order correlations following a multivariable linear regression analysis between environmental variables and bacterial abundance at the genus level. **a**. Maximum pain score with movement (first 24 h) and the genus *Collinsella*. **b**. Maximum pain score with movement (first 24 h) and the genus *Coprobacter*. **c**. Analgesic consumption (morphine equivalence, first week) and the genus *Dialister*.

Maximum pain score was inversely correlated with the genera *Collinsella* (*p* = 0.0087, r^2^ = -0.671, present in 21 of 21 samples) and *Coprobacter* (*p* = 0.024, r^2^ = -0.490, present in 14 of 21 samples). Analgesic consumption was positively correlated with the genus *Dialister* (*p* = 0.036, r^2^ = 0.439, present in 23 of 23 samples). However, these associations were not present after correcting for the multiple variables of age, Groupings 1, 2, maximum pain score with movement (first 24 h), and analgesic consumption (morphine equivalence, first week) **(**Fig. 5**)**.

**Fig. 6:**
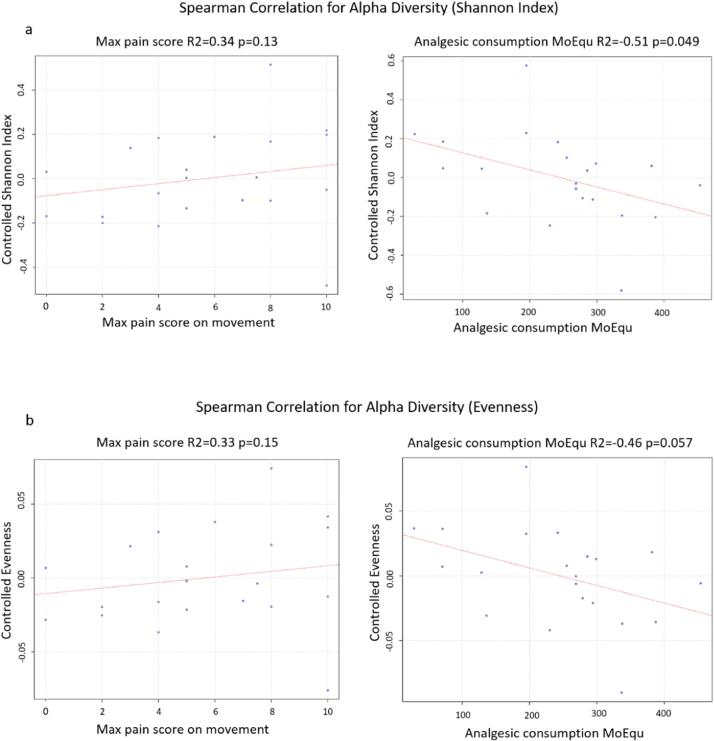
A multivariable linear regression model with Spearman correlation was used to determine any association between: **a.** The Shannon index of alpha diversity against Maximum pain score with movement (first 24 h) and Analgesic consumption (morphine equivalence, first week). **b.** The Evenness metric of alpha diversity against Maximum pain score with movement (first 24 h) and Analgesic consumption (morphine equivalence, first week).

Analgesic consumption was inversely correlated with the Shannon index of alpha diversity (overall taxa diversity) (*p* = 0.0499, r^2^ = 0.51). **(**Fig. 6**).** There were no other significant differences noted for other measures of alpha diversity.

## 4 Discussion

Most patients (17/20) experienced RP after resolution of ABPB. The magnitude of the RP, pain experience during the first week postoperatively and the analgesic consumption varied greatly between patients. Pain perception was associated with the abundance of certain genii, including Collinsella. We found no correlation or association between the magnitude of RP and (i) preoperative pain scores, (ii) type of the surgery, (iii) analgesic consumption, (iv) adherence to analgesic protocol (v) demographic data and (vi) QST results.

A major finding of this study is that postoperative analgesic consumption was inversely correlated with the Shannon index of alpha diversity. It is known that alpha diversity is decreased in certain pain conditions e.g. IBS and chronic pelvic pain syndrome (Cruz-Aguliar et al., 2019, Shoskes et al., 2016). With respect to the therapeutic potential of the gut microbiota, in IBS patients, symptoms (primarily abdominal pain) are decreased after FMT. This benefit is associated with an increase in alpha diversity of microbiota after FMT as well as the relative abundance of Akkermansia muciniphila being inversely correlated with pain reduction (Cruz-Aguliar et al., 2019). However it is worth noting, in patients with symptomatic diverticular disease, faecal calprotectin (a non-specific measure of disease activity) levels are positively correlated with alpha diversity (Kvasnovsky et al., 2018). This inconsistency may be the consequence of methodological differences in measuring the active microbial community (transcribed 16S rRNA counts) vs. the total microbial community (16S rRNA gene) (Moen et al., 2018).

In our study LEfSe analysis demonstrated an inverse correlation between abundance of *Collinsella* and maximum VRS with movement. Collinsella has previously been shown to influence production of the pro-inflammatory cytokine IL-17A; its role in altering gut permeability and disease severity was confirmed in experimental arthritis (Chen et al., 2016). However, our small sample size and the complexity of intestinal permeability regulation, microbiome-gut-brain axis, immune- and pain processing systems may account for these apparently contradictory results.

We demonstrated greater abundance of Lachnospira and Alistipes in patients whose pain was perceived as “not acceptable”. This is consistent with other studies in which patients with migraine or healthy subjects with intestinal bloating had greater abundance of Lachnospira; also, IBS patients had increased abundance of the genus Alistipes (Bai, 2019, Jalanka-Tuovinen et al., 2011, Saulnier et al., 2011). Analgesic consumption in our study was positively correlated with abundance of Dialister, a genus previously shown to correlate with ankylosing spondylitis disease activity score (Tito et al., 2017).

Regarding “protective bacteria”, we demonstrated that Porphyromonas was more abundant in patients with acceptable pain levels. Such an anti-nociceptive effect of Porphyromonas gingivalis lipopolysaccharide has been shown previously in a preclinical model (Khan et al., 2019).

The existence of a potential pathway through which gut microbiota could affect pain perception is only hypothetical at this stage. One possible mechanism is disruption of bacterial balance/homeostasis leading to induced chronic low grade inflammation (van den Munckhof et al., 2018). There is a reciprocal relationship between the gut microbiota and the host immune system whereby this immune system-microbiota partnership presides over both protective responses to pathogens and maintenance of regulatory pathways (Belkaid and Hand, 2014). However, in instances where one system is compromised due to stress, age, overuse of antibiotics and/or changes in diet, the balance may be tipped in favour of disorders/symptoms associated with immune dysregulation. Targeting the gut microbiota through the use of prebiotics and probiotics is being considered as therapeutic interventions for inflammatory conditions which may have implications in pain management (Tsai et al., 2019).

According to Tighe and colleagues, the most important determinants of postoperative pain experience are the type of the surgery, age and gender (Tighe et al., 2015); however genetic and psychosocial factors, preoperative pain status and medication history (opioids, SSRIs, etc.)(Parthipan et al., 2019) are also relevant. We suggest that our preliminary findings reported here offer an important new avenue for understanding the determinants of postoperative pain.

There are several limitations of our study. The small sample size limits the confidence with which any definitive conclusions can be drawn. One of our aims was to establish a feasible investigation pathway (data collection, analysis, etc.) for future similar studies. Secondly, our sample was inhomogeneous in terms of (i) the days spent between injury and surgery, (ii) surgery type (K-wiring vs ORIF) and surgeon. Finally, we were unable to collect sufficient pre-operative stool to justify a second analysis relating to acute changes in gut microbiome composition *peri*-operatively.

We have demonstrated here that certain characteristics of gut microbiome composition and diversity are associated with the patient-reported magnitude of postoperative pain and analgesic consumption. We suggest that these findings justify further work to improve our understanding of the influence of the gut microbiome on postoperative pain, and potentially to identify new analgesic modalities in the future.

## CRediT authorship contribution statement

**D. Brenner:** Conceptualization, Data curation, Formal analysis, Investigation, Methodology, Project administration, Writing - original draft, Writing – review & editing. **P. Cherry:** Data curation, Formal analysis, Software, Visualization, Writing – review. **T. Switzer:** Investigation, Project administration, Writing - review & editing. **I. Butt:** Investigation, Project administration, Writing - review & editing. **C. Stanton:** Data curation, Formal analysis, writing - review & editing. **K. Murphy:** Data curation, Formal analysis, Software, Visualization, Writing - review. **B. McNamara:** Conceptualization, Resources, Writing - review & editing. **G. Iohom:** Conceptualization, Supervision, Writing - review & editing. **S. O'Mahony:** Conceptualization, Data curation, Formal analysis, Investigation, Methodology, Resources, Supervision, Writing - original draft, Writing – review & editing. **G. Shorten:** Conceptualization, Data curation, Formal analysis, Investigation, Methodology, Resources, Supervision, Writing - original draft, Writing – review & editing.

## Declaration of Competing Interest

This research did not receive any specific grant from funding agencies in the public, commercial, or not-for-profit sectors. George Shorten is a funded investigator, Insight II SFI Research Centre.
