# Supplementary material for: Pain after upper limb surgery under peripheral nerve block is associated with gut microbiome composition and diversity
Source: Neurobiol Pain. 2021 Aug 18;10:100072. doi: 10.1016/j.ynpai.2021.100072 (PMC8404729; doi:10.1016/j.ynpai.2021.100072)
Supplement: Supplementary data 1 [file mmc1.doc]

**Supplementary Figure 1.** Patient flow diagram

**Analysis**

**Enrollment**

**Follow up**

Assessed for eligibility (n=46)

Excluded (n=26)

♦  Not meeting inclusion criteria (n=17)

♦  Declined to participate (n=6)

♦  Other reasons (n=3)

- Technical difficulties (n=3)
- **Clinical** data analysed (n=20)
- **Gut microbiome** data analysed (n=18)
  - Excluded from analysis (breach of protocol in sample collection and storage) (n=2)
- Had **postoperative QST** (n=19)
- Gave **postoperative stool sample** (n=19)
- Filled **Short Form McGill Pain Questionnaire** (n=19)
- Filled **postoperative pain diary for 1 week** (n=20
- One month **follow up call** (n=20)
- Gave **preoperative stool sample** (n=5)
- Filled **preoperative pain diary** (n=20)
- Had **preoperative QST** (n=20)

Recruited (n=20)

Underwent planned surgery (n=20)
