# Supplementary material for: Pain after upper limb surgery under peripheral nerve block is associated with gut microbiome composition and diversity
Source: Neurobiol Pain. 2021 Aug 18;10:100072. doi: 10.1016/j.ynpai.2021.100072 (PMC8404729; doi:10.1016/j.ynpai.2021.100072)
Supplement: Supplementary data 2 [file mmc2.docx]

**Supplementary Figure 2:** Bacterial relative abundance at the Phylum and Family taxonomic levels.

**
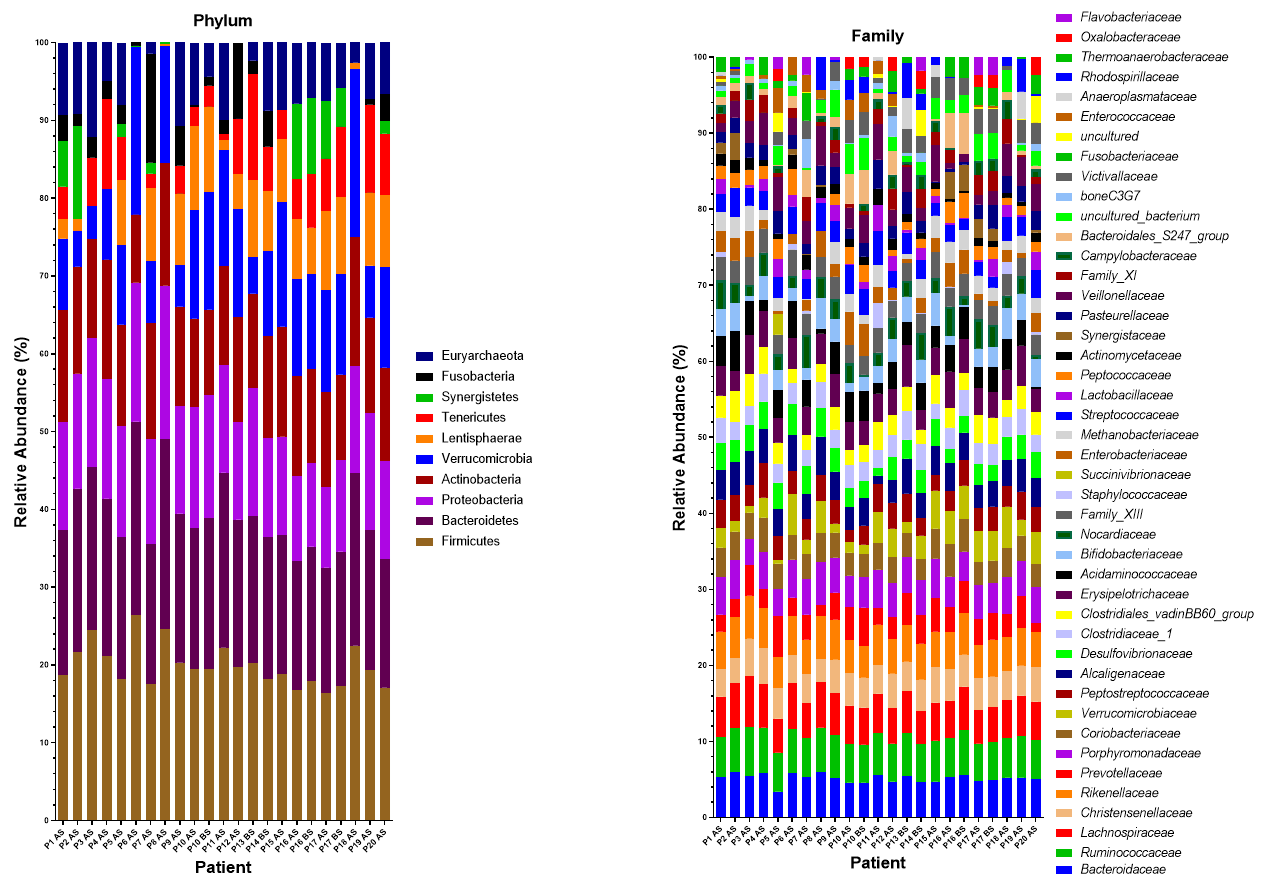
**
