# Supplementary material for: Pain after upper limb surgery under peripheral nerve block is associated with gut microbiome composition and diversity
Source: Neurobiol Pain. 2021 Aug 18;10:100072. doi: 10.1016/j.ynpai.2021.100072 (PMC8404729; doi:10.1016/j.ynpai.2021.100072)

**Supplementary Figure 3:** Heatmap containing the top 50 most abundant bacterial genera with > 0.1% relative abundance across all patients. Individuals are clustered based on Group 1 - Pain level acceptable to patient (first 24 hrs) - Yes/No.


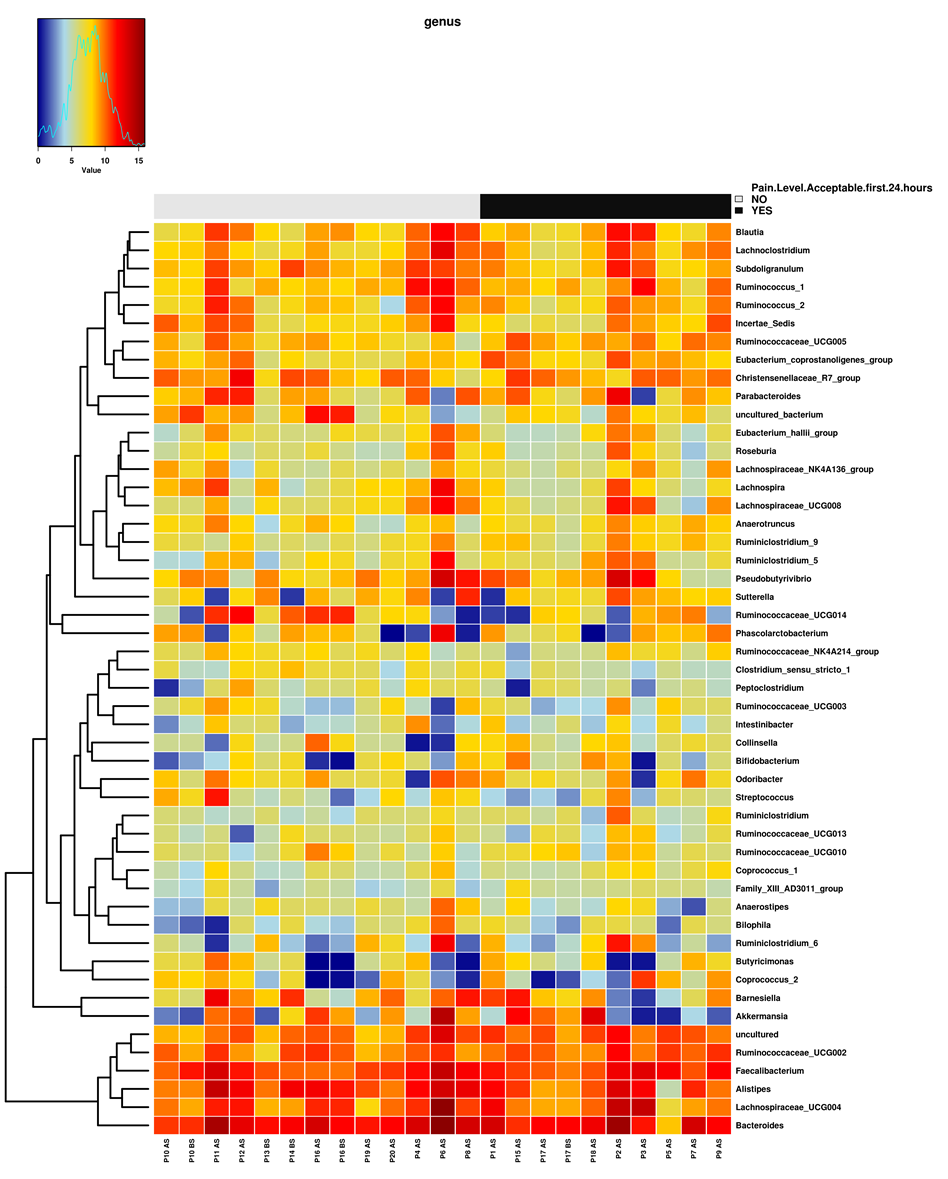

Supplement: Supplementary data 3 [file mmc3.docx]
