# Supplementary material for: Pain after upper limb surgery under peripheral nerve block is associated with gut microbiome composition and diversity
Source: Neurobiol Pain. 2021 Aug 18;10:100072. doi: 10.1016/j.ynpai.2021.100072 (PMC8404729; doi:10.1016/j.ynpai.2021.100072)
Supplement: Supplementary data 4 [file mmc4.docx]

**Supplementary Table 1.** Patient characteristics.

| Age (years) | 50.80 ± 13.34 |
| --- | --- |
| Gender F/M | 13 / 7 |
| ASA I/II | 11 / 9 |
| Weight (kg) | 75.44 ± 15.86 |
| Surgery ORIF / KW | 16 / 4 |
| Block failure | 0 |
| Total screened | 46 |
| Days between injury and surgery | 5.35 ± 4.19 |
| Days between surgery and postoperative stool sample | 2.94 ± 2.27 |
| Preoperative pain relief (yes/no) | 17/3 |
| Paracetamol and/or NSAIDs | 17/3 |
| Opiate | 11/9 |
| Dose (24 hour morphine equivalent, mg) | 13.8 (5.17 - 31.12) |

Values are expressed as numbers, mean ± SD or median (interquartile range) as appropriate.
