# Supplementary material for: Pain after upper limb surgery under peripheral nerve block is associated with gut microbiome composition and diversity
Source: Neurobiol Pain. 2021 Aug 18;10:100072. doi: 10.1016/j.ynpai.2021.100072 (PMC8404729; doi:10.1016/j.ynpai.2021.100072)
Supplement: Supplementary data 5 [file mmc5.docx]

**Supplementary Table 2.** QST results and rebound pain.

|  | | | **No RP (n=3)** | **RP (n=14)** | **Significance** |
| --- | --- | --- | --- | --- | --- |
| PPT | Surgical side | Preop | 63,3 (22,5) | 60,4 (26,5) | 0,86 |
|  |  | Postop | 94,6 (64,6) | 71 (34,1) | 0,36 |
|  | Control side | Preop | 67 (21,1) | 50,8 (22,8) | 0,27 |
|  |  | Postop | 59,6 (25,7) | 60,4 (36,5) | 0,97 |
|  |  |  |  |  |  |
| PTT | Surgical side | Preop | 84,3 (16,5) | 99,2 (54,3) | 0,65 |
|  |  | Postop | 115,3 (62,1) | 115,7 (68,3) | 0,99 |
|  | Control side | Preop | 97 (40,7) | 95,8 (57,6) | 0,97 |
|  |  | Postop | 87,6 (37,8) | 114,8 (89,9) | 0,62 |

Data expressed as mean (SD).
